# A new species of *Atheroides* Haliday (Hemiptera, Aphididae) native to North America

**DOI:** 10.3897/zookeys.452.8089

**Published:** 2014-11-04

**Authors:** Gary L. Miller, Andrew S. Jensen, Mark A. Metz, Robert R. Parmenter

**Affiliations:** 1 USDA, ARS, Systematic Entomology Laboratory, Beltsville, Maryland, USA; 2 Department of Entomology, Washington State University, Pullman, Washington, USA; 3 Scientific Services Division, Valles Caldera National Preserve (USDA), Jemez Springs, New Mexico, USA

**Keywords:** Valles Caldera National Preserve, Nearctic, cladistics, new species, *Atheroides*, Aphididae, Chaitophorinae, Siphini

## Abstract

We report and describe the first species of *Atheroides* Haliday presumed to be native to North America, collected at the Valles Caldera National Preserve, New Mexico, USA. We hypothesize its placement among the Siphini based on morphological, phylogenetic analysis and extend the distribution of the genus to the Holoarctic. We expand the key of the known *Atheroides* to include the new species and discuss the current hypotheses of the geographic distribution of the type species, *Atheroides serrulatus* Haliday.

## Introduction

The Jemez Mountains in northern New Mexico, USA, constitute a “sky island” at the southern end of the Rocky Mountains of North America. This area now serves as a Pleistocene biological refugium, supporting high-elevation ecosystems left behind by the last retreating Ice Age 20,000 years ago (Goff 2009). Increasing aridity during the last 10,000 years has created arid grassland valleys around the Jemez Mountains, isolating the montane forested and meadow ecosystems from similar habitats in adjacent mountain ranges. In the center of the Jemez Mountains lies a super-volcano’s caldera, which today encompasses the Valles Caldera National Preserve (VCNP) under the U.S. Department of Agriculture. As part of an inventory of the Preserve’s natural resources, VCNP scientists and entomologists from the USDA’s Systematic Entomology Laboratory began an extensive survey of the forests and *valles*. While producing numerous new distribution records for the state of New Mexico, no new insect taxa endemic to this ecosystem had been found. This work constitutes one of the first published accounts of a new species of insect collected at VCNP and unknown from any other locality. Remarkably the species is in a genus of aphids, *Atheroides* Haliday, 1839, with no known native, New World species. Because *Atheroides* prior to this discovery appeared to have a Palaearctic distribution except for the North American adventive, *Atheroides serrulatus*, this account may represent the first support for a geologic refuge.

*Atheroides* is part of the tribe Siphini Mordvilko, 1928, the most derived clade of the Chaitophorinae Mordvilko, 1909, and was treated extensively by Wieczorek (2009, 2010) and Wieczorek and Kajtoch (2011). The genus is considered to have a Palaearctic distribution. Although, the type species, *Atheroides serrulatus* Haliday, 1839, has been collected in Canada, the remaining species are known only from the Palaearctic region. Because of this apparent disjunct distribution *Atheroides serrulatus* was considered an adventive, albeit non-invasive, species to the New World (Richards 1972, Foottit and Richards 1993, Foottit et al. 2006). Compared with other members of Siphini, *Atheroides* can be distinguished by their elongate, narrow body and a semicircular tergite VIII that covers the cauda (Wieczorek 2010). Species of *Atheroides* are known to feed on grasses and sedges. Their long, flat bodies seem ideal for positioning themselves between blades of grass, and the blunt, apical segment of the rostrum is diagnostic for grass-feeding aphids (Wieczorek 2009). Species in the genus also live singly or in small colonies (Wieczorek 2009), so considering their semi-reclusive, inconspicuous habits there may be more species awaiting discovery. In this paper we report and describe the first species presumed to be native to North America; hypothesize its placement among the Siphini based on morphological, phylogenetic analysis; and unquestioningly expand the distribution of the genus to Holoarctic.

## Methods

We follow the recommendations of Thompson and Mathis (1980) for the names of Haliday first appearing in Curtis 1837 in terms of availability, validity, and priority. After DNA extractions were obtained, we mounted the specimens in Canada balsam (Favret 2005) and deposited them in the U.S. National Aphid collection, located at the Henry A. Wallace Beltsville Agricultural Research Center, Beltsville, Maryland, USA.

We made observations with both stereo and compound microscopes at magnifications from 100-400×. We used a Visionary Digital BK Lab System® and SolMate® Trans-Illumination System to take digital photographs of slide-mounted specimens with a Canon® EOS 5D Mark II DSLR camera. We montaged Z-stacks with Helicon Focus Pro®. Illustrations were first hand-drawn using a Nikon® Eclipse E600 and a drawing tube. Final illustrations were rendered on Denril® multi-media vellum using Pigma® Micron® 005 and 01 technical pens. We performed digital image editing/enhancement/manipulation in the Gnu Image Manipulation Program (GIMP). Dimensions of structures in the description are reported in millimetres; for apterous viviparae the first measure is that of the holotype and the second that of the paratype; for oviparae ranges are followed by means in parentheses.

We approached the phylogenetic analysis with the assumptions that Siphini was monophyletic and part of the Chaitophorinae, that the species in question was undescribed, and that the new species belonged to Siphini. The phylogenetic question was the correct generic placement of the new species. We sampled taxa within Siphini so as to have all of the type species of the currently included genera (Wieczorek 2010; Wieczorek and Kajtoch 2011), and chose as the outgroup taxon the type species of Chaitophorinae. We included two species of each subgenus of *Sipha*, since support of a monophyletic *Sipha* requires further research (Wieczorek 2010; Wieczorek and Kajtoch 2011). Our entire taxon sample for testing the placement of the new species was: *Chaitophorus leucomelas* Koch, 1854 (type of Chaitophorinae); *Atheroides serrulatus* (type of *Atheroides*); *Laingia psammae* Theobald, 1922 (type of *Laingia* Theobald, 1922); Sipha (Rungsia) maydis Passerini, 1860 (type of the subgenus *Rungsia* Mimeur, 1933); Sipha (Rungsia) elegans Del Guercio, 1905; *Chaetosiphella berlesei* (Del Guercio, 1905) (*Sipha*) (type of *Chaetosiphella* Hille Ris Lambers, 1939); Sipha (Sipha) glyceriae (Kaltenbach, 1843) (*Aphis* L., 1758) (type of *Sipha*); Sipha (Sipha) flava (Forbes, 1884) (*Chaitophorus* Koch, 1854); *Caricosipha paniculata* Börner, 1939 (type of *Caricosipha* Börner, 1939). We included in the cladistic matrix characters used previously by Wieczorek (2010) and Wieczorek and Kajtoch (2011) (Characters 1, 3, 5, 8, 9, 10, and 11 in this analysis), but could not use those author’s other characters because they were either uninformative among the current taxa sampled, were for characters of other life stages and/or sexes, or we could not score them unambiguously. We considered the coded states of their character 7 (“dorsal cuticle: (0) reticular or spinulose structures present; (1) smooth”) to be not homologous, so separated those features into two separate characters (Characters 6 and 7 in this analysis.). We introduced characters 0, 2, and 4. As a part of character mining, we measured all body segments, leg segments, antennal segments and rostral segments on the exemplars of all taxa, and used these measures to calculate every combination of ratios among them. We plotted the distribution of the calculated ratios among all taxa on histograms to check for bi- or multimodal distributions. We removed all characters we could not score unambiguously or had overlap in quantitive measures/ratios. We weighted all characters equally and coded them as unordered. We preferred combinations of binary characters and nominal state coding over multistate characters. We performed preliminary phylogenetic analysis using the Parsimony Ratchet routine in Winona (5000 iterations per rep, 5 trees held per iteration, 1 character sampled, and remaining parameters set to default; Nixon 1999). We did not need multiple reiteration on a dataset of this size. We then conducted exhaustive searches (implicit enumeration) using TNT (Willi Hennig Society edition; Goloboff et al. 2008) to corroborate the preliminary tree hypothesis. We calculated Bremer values (Bremer 1988) in TNT from exhaustive searches of progressively longer suboptimal trees (increments of 1 step).

We stored the HT apterous adult female and two oviparous adults collected in Valles Caldera National Preserve in 95% ethanol at -20 °C, until the DNA extractions were performed. We extracted DNA non-destructively with Qiagen®’s Blood & Tissue kits (Valencia, CA) using a technique described by Favret (2005). The extractions followed the “Purification of Total DNA Animal Tissues” protocol with a few modifications (Qiagen 2006). Firstly, instead of the whole insect being pulverized, we pierced the integument of the aphid using a minuten insect pin. Secondly, we allowed the initial cell lysis step to continue for 24 hours instead of the recommended 1–3 hours. This extra time allowed the specimens to clear for microscope slide mounting. We amplified the barcoding region of COI using the forward primer: C1-J-1490: 59-ATTCAACCAATCATAAAGATATTGG-39 and reverse primer: C1-N-2198: 59-TAAACTTCTGGATGTCCAAAAAATCA-39 (Hajibabaei et al. 2006). We sequenced the fragment of COI in both directions using these primers with Applied Biosystems BigDye® kits, version 3.1, and read on an Applied Biosystems® sequencer. We assembled and aligned sequences with Sequencher® v. 4.7.

**Figure 1.**
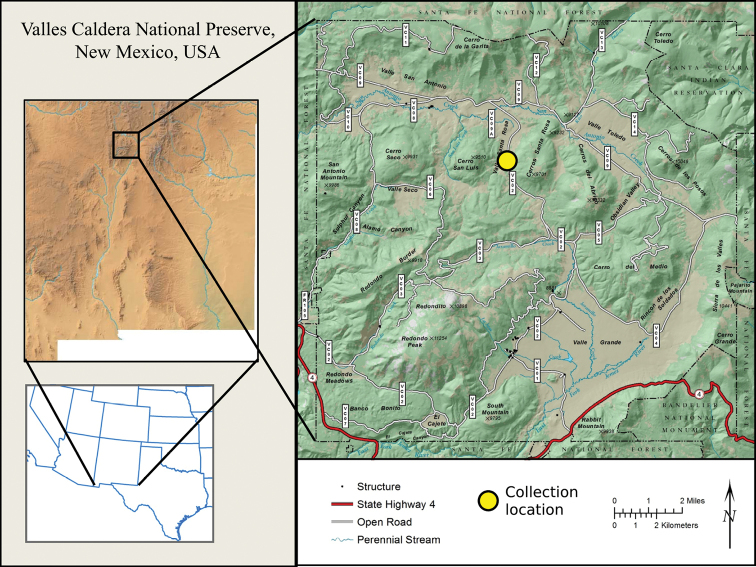
Map of the Valles Caldera National Preserve, New Mexico, USA, showing collection location.

**Figure 2.**
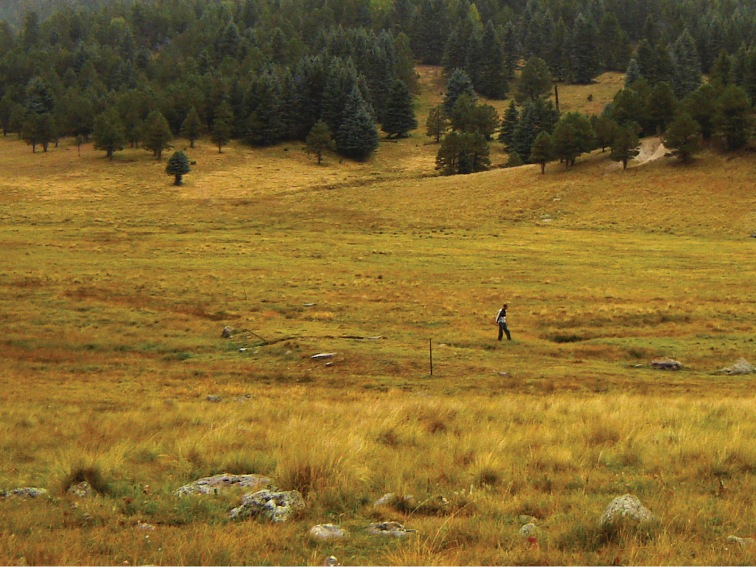
Second author in the habitat of the type locality for the new species along Santa Rosa Creek.

## Results

We were able to resolve a total of 12 binary morphological characters to address the question of generic placement of the new species among the genera of Siphini. Description of characters and character states included in the analysis follow:

0. Ratio of the Entire Body Length to the Body Width Measured Side to Side Across the Siphunculi

0 Body length less than 3× width of body

1 Body length at least 3.5× width of body

Wieczorek (2009) diagnosis *Atheroides* as “elongate, slender, nearly linear” (except *Atheroides brevicornis* Laing, 1920). We used a ratio of length to width to quantify the elongation and narrowness of the taxa since the total length among the taxa investigated does not vary considerably. The length of the body was taken from the frons to the terminus of tergite VIII, and the width of the body was taken at the level of the siphunculi so as to make it objectively repeatable. The new species shares the derived state with *Atheroides serrulatus* and *Laingia psammae*.

1. Number of Antennal Segments

0 Six

1 Five

A five-segmented antenna has long been considered a diagnostic feature of Siphini among aphid workers and was corroborated by Wieczorek (2010) through cladistic analysis. The new species shares the derived state with all the species of Siphini included in the analysis.

2. Ratio of Length of Dorsad Apical Seta of Antennal Segment III to Width of Antennal Segment III Measured at Middle

0 Seta length at least 1.3× width of antennal segment 3 at middle

1 Seta absent or seta length subequal or less than width of antennal segment 3

The new species is notable among Siphini in having short, sparse setae on the antenna, particularly antennal segment III. We used a ratio of the length of the most apical dorsal seta on antennal segment III to the width of antennal segment III at the middle of the segment since that width did not vary considerably among the taxa investigated. The new species shares the derived state with *Atheroides serrulatus*.

3. Ratio of the Length of the Processus Terminalis to the Base of the Terminal Antennal Segment

0 Processus terminalis shorter or subequal in length to the base of the terminal antennal segment

1 Processus terminalis at least 1.5× longer than the base of the terminal segment

The ratio of the lengths of the base of the terminal antennal segment and the processus terminalis is used extensively among aphid taxa for genus and species level identification. This character is stable among taxa in the analysis − autopomorphic for *Chaetosiphella berlesei* − so is uninformative in this tree hypothesis, but is left in for the benefit of future work in the group.

4. Ratio of Hindfemur Length to Midfemur Length

0 Hindfemur length less than 1.5× length of midfemur

1 Hindfemur length at least 1.6× length of midfemur

The new species is notable among Siphini in having a short femur on the fore- and midlegs, particularly as compared to the femur of the hindlegs. The new species shares the derived state with *Atheroides serrulatus*.

5. Shape of the Setae on the Dorsum of the Body

0 Dorsal setae almost exclusively acuminate

1 Dorsal setae scale-like, denticulate, and/or flabellate

The new species shares with other species of *Atheroides* the presence of non-acuminate setae throughout the body surface. The setae of the dorsum are characteristic of this diagnostic feature. The new species shares the derived state with *Atheroides serrulatus*.

6. Denticulate or Spiculate Cuticle Covering Most of Body

0 Absent

1 Present

The integument between setal sockets may have raised, densely-distributed extensions that can be acuminate or blunt, as opposed to smooth cuticle. The derived state of this character does not support any monophyletic group resolved in the tree hypothesis.

7. Dorsal Cuticular Surface Wrinkles and Folds

0 Absent

1 Present

The new species shares with other species of *Atheroides* the presence of wrinkles and/or folds throughout the dorsal cuticle of the body reminiscent of the surface of the human brain. The new species shares the derived state with *Atheroides serrulatus*.

8. Position of the Base of the Siphunculi

0 On abominal segment VI

1 On abdominal segment V

The primitive state among Aphididae is for the siphunculi to be positioned on tergite VI. Among most Siphini the position of the siphunculi is on tergite V. The tree hypothesis created from this analysis suggests that the derived state supports the monophyly of Siphini sans *Caricosipha paniculata* with a reversal in *Laingia psammae*.

9. Orifice of Siphunculus

0 Elevated above the surface of the abdomen on a tube

1 Flush with the surface of the abdomen or on a short mound of cuticle, not elevated by a tube

The primitive state among Aphididae is for the siphunculous to be in the form of a tube or cylinder such that the external orifice is elevated above the surface of the dorsum. The new species shares the derived state with all the species of Siphini included in the analysis except *Caricosipha paniculatae*.

10. Posterior Margin of Tergite VIII

0 Not expanded posteriorly, cauda visible in dorsal view

1 Expanded posteriorly hiding cauda mostly or completely from dorsal view

The new species shares with the derived state with *Atheroides serrulatus* and *Laingia psammae*.

11. Shape of cauda

0 Constricted basally or subbasally creating an apical knob

1 Broadly rounded, truncate, or emarginate

The new species shares with other species of *Atheroides* the shape of the cauda, being broadly rounded, truncate, or emarginate as opposed to having a posterior elongation or a constriction with an apical knob. The new species shares the derived state with all the species of Siphini included in the analysis except *Caricosipha paniculatae* and the two species of Sipha (Sipha).

**Figure 3.**
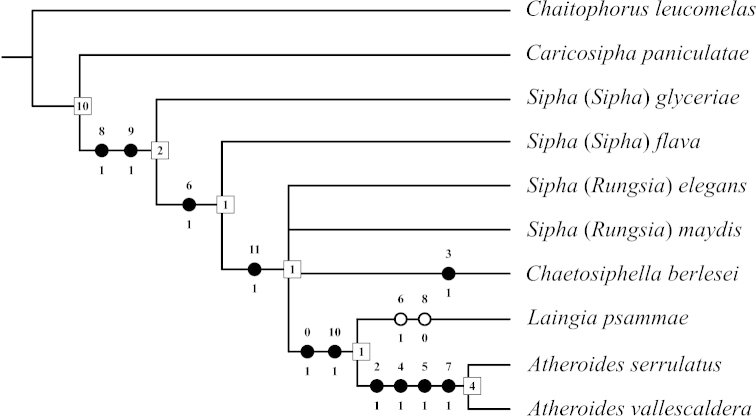
Single, most parsimonious tree of 14 steps (CI=85, RI=88) resulting from an exhaustive search in TNT. Closed circles indicate unique forward changes. Open circles indicate either forward changes with homoplasy or reversals. Numbers on nodes in squares indicate Bremer support values for that node.

There were no missing or inapplicable characters in the matrix. The complete matrix is given in Table 1. An exhaustive tree search resulted in a single, most parsimonious hypothesis of 14 steps (CI=85, RI=88) (Fig. 3). There is no character evidence to support relationships among the two species of Sipha (Rungsia) and *Chaetosiphella berlesei* so we collapsed these nodes (Fig. 3). The data support a monophyly including the new species and *Atheroides serrulatus*, the type species of *Atheroides*, (Bremer support of 4; Fig. 3).

**Table 1.** Characters matrix.

	Character											
Taxon	0	1	2	3	4	5	6	7	8	9	10	11
*Chaitophorus leucomelas* Koch, 1854	0	0	0	0	0	0	0	0	0	0	0	0
Sipha (Rungsia) elegans Del Guercio, 1905	0	1	0	0	0	0	1	0	1	1	0	1
Sipha (Rungsia) maydis Passerini, 1860	0	1	0	0	0	0	1	0	1	1	0	1
Sipha (Sipha) flava (Forbes, 1885)	0	1	0	0	0	0	1	0	1	1	0	0
Sipha (Sipha) glyceriae (Kaltenbach, 1843)	0	1	0	0	0	0	0	0	1	1	0	0
*Caricosipha paniculatae* Börner, 1939	0	1	0	0	0	0	0	0	0	0	0	0
*Laingia psammae* Theobald, 1922	1	1	0	0	0	0	0	0	0	1	1	1
*Chaetosiphella berlesei* (Del Guercio, 1905)	0	1	0	1	0	0	1	0	1	1	0	1
*Atheroides vallescaldera* **sp. n.**	1	1	1	0	1	1	1	1	1	1	1	1
*Atheroides serrulatus* Haliday, 1839	1	1	1	0	1	1	1	1	1	1	1	1

We amplified and sequenced a 652 bp DNA fragment containing the barcoding region of the mitochondrial COI gene from the HT and two oviparous vouchers. Since all individuals produced sequences that were identical, only one sequence was submitted to GenBank (accession number KJ737374). We searched for matching sequences in GenBank and found multiple matches for Aphididae, but there were no sequences available for the taxa sampled in the phylogenetic analysis with enough overlap to warrant inclusion.

### Atheroides

Taxon classificationAnimaliaHemipteraAphididae

Haliday, 1839

Atheroides Haliday 1837: 218 (Nomen nudum)
Atheroides Haliday, 1839: 189. Type species *Atheroides serrulatus* Haliday, 1839: 189 by subsequent designation (Kirkaldy 1906: 10) (Note: Laing (1920) incorrectly designates *Atheroides serrulatus* Haliday as the type species again subsequent to Kirkaldy).
Apteroides Mordvilko, 1929: 91. (Subsequent misspelling)
Corealachnus Paik, 1971: 3. Type species *Corealachnus suwonensis* Paik, 1971: 4 by original designation.

### Atheroides vallescaldera

Taxon classificationAnimaliaHemipteraAphididae

Miller & Jensen sp. n.

http://zoobank.org/DBF1845E-8DBC-4B1B-920B-4AD9F9197FF6

#### Diagnosis.

This new species can be distinguished from other species in the genus by the following combination of characters: setae on the dorsum flabellate and dentate, and arranged in rows; dorsum sclerotized with rugose sculpturing; marginal setae of abdominal tergites I-VI easily visible, longer than width of hindfemur at middle, acuminate; empodial setae flat, but with base and apex of equal width, not spatulate.

#### Description

**Figures 4–11.**
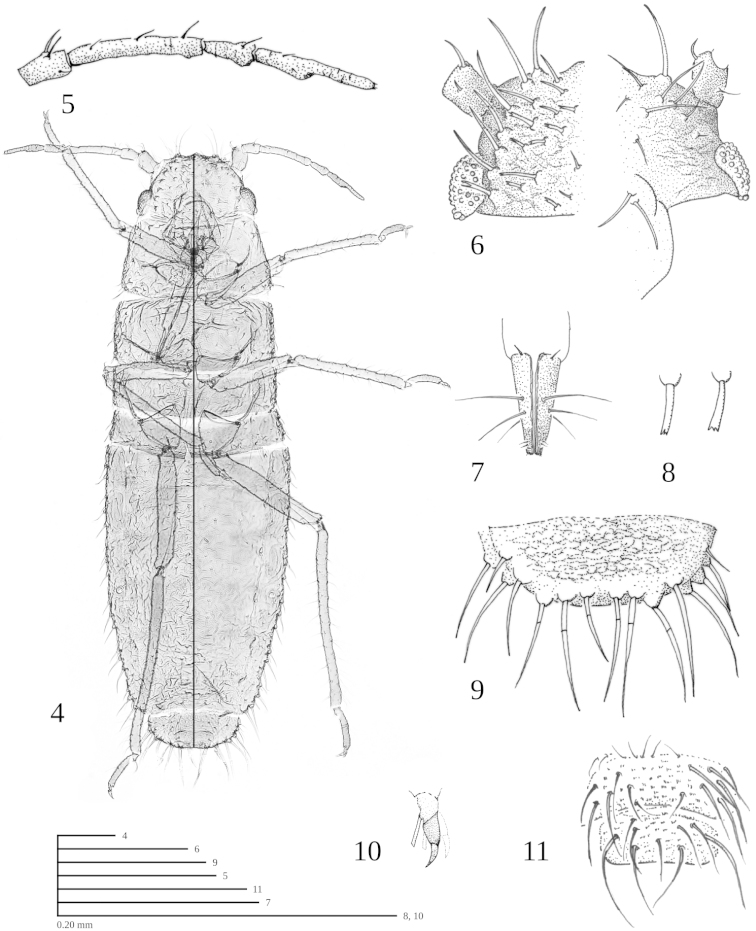
*Atheroides vallescaldera* n. sp. **4** holotype habitus (Left side focal planes from dorsum to middle. Right side focal planes from middle to ventral surface.) **5** antenna of apterous vivipara **6** head (left half dorsal, right half ventral) **7** ultimate rostral segments **8** examples of dorsal abdominal setae variation **9** tergite VIII (dorsal) **10** empodial setae **11** anal and genital figs. (Scale indicated by 0.20 mm measure bars and corresponding figure number.).

(slide-mounted specimens). *Apterous vivipara* (Figs 4–11, Table 2) (n = 2): Body at least 3 times longer than wide, dorsum rugose, dorsal setae on segments I-VII 0.018–0.058, mostly dentate and flabellate with some acuminate, arranged in rows, marginal setae acuminate, longer than width of hindfemur at middle, present on all abdominal segments. Head (Fig. 6) rectangular, flattened dorsoventrally and frons flat with bluntly pointed projections, one medial projection more prominent, rugose; setae acuminate on front and sides and denticulate (Fig. 8) dorsally; antennal tubercle undeveloped, basal antennal articulation flush with side of head; eyes slightly inset, eye outer margin almost flush with head margin, sometimes partially obscured dorsally, triommatidium outer margin projecting only as far as eye outer margin. Antenna (Fig. 5) 5-segmented, not reaching hind margin of prothorax, without secondary sensoria, antennal setae length usually subequal to width of corresponding antennal segment or less at middle of segment. Ultimate rostral segment (Fig. 7) with 2 pairs of primary setae and 1 pair of secondary setae. Basitarsi with 1 stout spine and 4 acuminate setae, one longer than basitarsus, the rest subequal to basitarsus; empodial setae flat, but base and apex of equal width, not spatulate (Fig. 10). Siphunculus flush with the surface of tergite V, without any elevation above the surface of the cuticle, orifice surrounded by a thickened band of cuticle. Tergite VIII (Fig. 9) broadly rounded, extending posteriorly so that it covers cauda, with robust, acuminate marginal setae. Cauda indistinct, obscured by setae of tergite VIII. Anal fig slightly emarginate, genital slightly emarginate (Fig. 11) with numerous irregularly arranged setae. Morphometric data are in Table 2.

**Table 2.** Morphometric data for apterous viviparae and oviparae of *Atheroides vallescaldera* sp. n.

	*Atheroides vallescaldera* sp. n.		*Atheroides serrulatus* (from Wieczorek 2009, 2010)	
	**Oviparae**	**Viviparae**	**Oviparae**	**Viviparae**
	**N=8**	**N=2**	**N=6**	**N=17**
Body [mm]	2.08–2.30	2.07–2.21	2.00–2.35	1.70–2.20
Antenna [mm]	0.54–0.58	0.53–0.55	-	-
Antenna / Body [times]	0.24–0.27	0.25–0.26	0.21	0.19
Ant. segm. III [mm]	0.17–0.19	0.17–0.18	-	-
Ant. segm. IV [mm]	0.06–0.08	0.07–0.07	-	-
Ant. segm. V base [mm]	0.07–0.09	0.08–0.08	-	-
Ant. Segm. V base / Ant segm. III [times]	0.41–0.53	0.44–0.49	0.50–0.55	0.50–0.70
Ant. segm. V processus terminalis [mm]	0.07–0.09	0.08–0.08	-	-
Ant. segm. V processus terminalis / Ant. segm. III [times]	0.39–0.53	0.43–0.45	-	-
Ant. segm. V processus terminalis / base [times]	0.77–1.29	0.89–1.03	1.00–1.05	0.77–0.91
Ant. Segm. V / Ant. Segm. III [times]	0.82–1.00	0.89–0.92	1.00–1.10	1.10–1.30
Ant. Segm. V / Ant. Segm. IV [times]	2.07–2.58	2.27–2.33	3.00–3.25	2.20–3.50
Ultimate rostral segm. [mm]	0.12–0.13	0.12–0.13	0.07–0.10	0.07–0.10
Ultimate rostral segm. / its basal width [times]	1.77–2.36	2.10–2.15	-	-
Ultimate rostral segm. / Ant. segm. V base [times]	1.43–1.79	1.50–1.53	-	-
Ultimate rostral segm. / Ant. segm. III [times]	0.66–0.76	0.67–0.73	0.69	0.69
Ultimate rostral segm. / Hind tarsus, 2nd segm. [times]	0.77–1.00	0.83-0.98	0.75	0.75
Hind femur [mm]	0.33–0.38	0.37–0.37	-	-
Hind tibia [mm]	0.55–0.65	0.62–0.62	-	-
Hind tibia / Body [times]	0.26–0.29	0.28–0.30	-	-
Hind tarsus, 2nd segm. [mm]	0.13–0.14	0.13–0.14	-	-
Siphunculus width [mm]	0.02–0.03	0.02–0.03	-	-

*DNA barcode* (COI) for the holotype and one paratype contains 652 nucleotides (GenBank # KJ737374):

GAACTTTATATTTTTTATTTGGAATTTGATCAGGACTAATTGGTTCTTCACTAAGAATTTTAATTCGATTAGAATTAAGACAAATTAATTCAATCATTAATAATAATCAATTATATAATGTTATCATTACAATTCATGCATTTATTATAATTTTTTTTATAACTATACCAATTGTAATTGGTGGATTTGGAAATTGATTAATCCCTTTAATAATAGGATGCCCTGATATATCATTCCCACGATTAAATAATATTAGATTTTGAATACTTCCACCAGCATTAATATTTATAATTATAAGTTTTATAATTAATAATGGAACAGGAACAGGATGAACAATTTACCCCCCTCTATCTAACAATATTGCCCATAATAATATTTCTGTTGACTTAACAATTTTTTCTCTACATTTAGCAGGAATCTCATCAATTTTAGGAGCAATCAATTTTATTTGCACAATTATAAATATAATACCTAATAATATAAAAATTAATCAAATTCCTCTTTTCCCTTGATCTATTTTAATTACAGCAATCTTATTAATTTTATCTTTACCTGTATTAGCAGGTGCAATTACTATACTTTTAACTGATCGAAATCTTAATACTTCATTTTTTGATCCTTCAGGAGGTGGAGACCCTATCTTGTATCAACA

*Alate vivipara and male*: unknown.

*Ovipara* (n = 8): Dorsal abdominal setae on segments I-VII 0.015–0.070. With 18–27 circular pseudosensoria on each hindtibia predominantly organized in pairs that are 8-shaped, others are single circles or triplets (conjoined pairs or triplets are still counted as single pseudosensoria). Otherwise similar to apterous vivipara. Morphometric data are in Table 2.

#### Etymology of specific epithet.

The specific epithet, *vallescaldera*, is derived from the locality in which the specimens were collected, the Valles Caldera National Preserve, and should be considered a compound noun in apposition.

#### Specimens examined.

**Type-locality.** USA: NEW MEXICO: Sandoval Co., near unit 12, lower Santa Rosa Creek watershed, a perennial stream tributary of the Rio San Antonio, 35.951; -106.521, VCNP# 144, 23.ix.2010, 2,595m, A. Jensen coll., ex grass in open meadow next to Santa Rosa Creek, dominated by sedges, grasses, rushes and a variety of forbs (Fig. 2).

Holotype apterous vivipara (Figs 4–11): Slide-mounted in balsam USNMENT 00826485.

Original label: “New Mexico: Valles Caldera Nat’l Preserve Near Unit 12 22 Sept. 2010 ex: grass in open meadow A. Jensen coll. SEL VCNP#144 Balsam”

Paratypes: same data as holotype (1 apterous vivipara USNMENT 00826486, 8 oviparae USNMENT 00826487-89, USNMENT 00826480-84).

#### Host plants and habitat.

Unknown bunch grass. All other known *Atheroides* spp. feed on a variety of grasses (Poaceae), sedges (Cyperaceae) and rushes (Juncaceae) (Wieczorek 2009). On the VCNP, there are 88 taxa of grasses, 31 taxa of sedges, and 11 taxa of rushes (includes subspecies and varieties). Among these plant taxa at the collection locale are common host plants of other *Atheroides* spp., including *Deschampsia caespitosa*, *Phleum* spp., *Festuca* spp., *Carex* spp., and *Juncus* spp.

### Key to the known species of *Atheroides* (apterous viviparae) (modified from Wieczorek 2009)

**Table:** 

1	Setae on the dorsum of the body exclusively acuminate	**2**
1’	Setae on the dorsum of the body acuminate, forked, dentate, and/or flabellate	**3**
2 (1)	Spinal setae very long, as long as or longer than marginal ones. Cauda covered by abdominal tergite VIII. On *Deschampsia caespitosa*	***doncasteri* Ossiannilsson, 1955**
2’	Marginal setae very long, longer than spinal ones. Cauda not covered by abdominal tergite VIII. On various grasses	***hirtellus* Haliday, 1839**
3 (2’)	Dorsum partially sclerotic without visible sculpture. Antennal segment III with 4–8 long setae. On *Festuca ovina* and *Stipa splendens*	***karakumi* Mordvilko, 1948**
3’	Dorsum sclerotic with visible, rugose sculpture. Antennal segment III with 0–4 short setae. On various grasses	**4**
4 (3’)	Dorsal setae arranged in visible rows	**5**
4’	Dorsal setae not arranged in visible rows	**6**
5 (4)	Marginal setae of abdominal tergites I–VI short, hardly visible, rarely as long as width of hindfemur, dentate. Empodial setae spatulate, flat and broadened at apex	***serrulatus* Haliday, 1839**
5’	Marginal setae of abdominal tergites I–VI easily visible, longer than width of hind femur at middle, acuminate (Fig. 4). Empodial setae flat, but with base and apex of equal width, not spatulate (Fig. 10)	***vallescaldera* sp. n.**
6 (4’)	Body elongate, oval, 1.50–2.40 mm long. Antennae 4- or 5-segmented, 0.12–0.15 times body length. Antennal segment I with 2 pointed and 1 dentate seta	***brevicornis* Laing, 1920**
6’	Body elongate, slender, nearly linear, 1.55–1.72 mm long. Antennae 5 segmented, 0.18–0.25 times body length. Antennal segment I with 1 erect fan-shaped seta	***persianus* Wieczorek, 2009**

### Key to the North American species of *Atheroides* (oviparae) (modified from Wieczorek 2010)

**Table:** 

1	Hind tibiae with more than 30 pseudosensoria. Marginal setae of abdominal tergites I-VI short, hardly visible, rarely as long as width of hind femur, dentate. Empodial setae spatulate, flat and broadened at apex	***serrulatus* Haliday, 1839**
1’	Hind tibiae with less than 30 pseudosensoria. Marginal setae of abdominal tergites I-VI easily visible, longer than width of hindfemur at middle, acuminate (Fig. 4). Empodial setae flat, with base and apex of equal width, not spatulate (Fig. 10)	***vallescaldera* sp. n.**

## Discussion

We have shown that *Atheroides vallescaldera* is morphologically most similar to *Atheroides serrulatus* among described *Atheroides*, yet there are clear morphological differences between the species. The facts that it is found in such an unusual geological feature as the Valles Caldera, and far from the known populations of *Atheroides serrulatus* in Canada, suggest that *Atheroides vallescaldera* is native to North America. This is a simpler explanation than the notion that two species of *Atheroides* have invaded North America, with one being unknown in the Palearctic yet establishing in an isolated and unique habitat in North America. Further, we suggest that it is possible that *Atheroides serrulatus* is naturally Holarctic as opposed to adventive in North America.

The discovery of a new *Atheroides* species in the course of general aphid collecting using a beating tray technique (i.e. not specifically targeting cryptic grass feeders such as *Atheroides*) suggests that directed searching for *Atheroides* in North America may lead to discovery of additional native species. Future field work should include accurate species identification of host plants, searching a range of habitats including extremes of altitude, latitude, and precipitation, and detailed notes on microhabitats.

## Supplementary Material

XML Treatment for Atheroides

XML Treatment for Atheroides vallescaldera
